# No major difference in perceived quality of care in patients with hip or knee osteoarthritis assessed in a physical therapy-led triage compared with standard care: a randomized controlled trial

**DOI:** 10.1186/s12891-023-06659-5

**Published:** 2023-06-29

**Authors:** Linnea Gustavsson, Maziar Mohaddes, Karin Samsson, Susanne Beischer

**Affiliations:** 1 Institute of Clinical Sciences, Department of Orthopaedics, Sahlgrenska Academy, University of Gothenburg, Gothenburg, Sweden; 2 Department of Orthopaedics, Sahlgrenska University Hospital, Gothenburg, Sweden; 3 Sportrehab, Gothenburg, Sweden; 4 Institute of Neuroscience and Rehabilitation, Department of Health and Rehabilitation, Sahlgrenska Academy, University of Gothenburg, Gothenburg, Sweden; 5 Capio Ortho Center Göteborg, Gothenburg, Sweden

**Keywords:** Advanced physiotherapy, Physical therapy, Quality of care, Patient perception, Expectations, Triage

## Abstract

**Background:**

Physical therapy-led orthopedic triage is a care model used to optimize pathways for patients with hip or knee osteoarthritis. However, scientific evidence of the effectiveness of this model of care is still limited and only a few studies report patients’ perception of it. The aim of this study was to compare patients’ perceived quality of care after physical therapy-led triage with standard practice in a secondary care setting for patients with primary hip or knee osteoarthritis.

**Methods:**

In this randomized study, patients with hip or knee osteoarthritis referred for an orthopedic consultation received either physical therapy-led triage (n = 344) or a standard care assessment by an orthopedic surgeon (n = 294). To evaluate the patients’ perceived quality of care, a short version of the *Quality from the Patient’s Perspective* (QPP) questionnaire was sent to the patients within a week after their assessment. The primary outcome was the statement *“I received the best examination and treatment” on QPP.*

**Results:**

A total of 348 patients (70%, physical therapy-led triage: n = 249, standard care: n = 199) answered the questionnaire. No significant difference was found in the primary outcome between the groups (*p* = 0.6). Participants in the triage group perceived themselves to have received significantly better information about how to take care of their osteoarthritis (*p* = 0.017) compared with the standard care group. The standard care group reported that they participated in the decision-making process to a greater extent (*p* = 0.005), that their expectations were met to a greater degree (*p* = 0.013), and that their care depended more on their need for care rather than the caregivers’ routines (0.007).

**Conclusion:**

Both groups report high perceived quality of care. Significant differences were found in four of 14 questions, one in favor of the physical therapist and three in favor of the standard care group. The findings of this study are in line with previous research and support the use of this care model for patients with hip or knee OA in secondary care. However, due to the dropout size, the results should be interpreted with caution.

**Trial registration:**

Clinical Trials NCT04665908, registered 14/12/2020.

**Supplementary Information:**

The online version contains supplementary material available at 10.1186/s12891-023-06659-5.

## Background

In Sweden and other European countries, patients with osteoarthritis (OA) are traditionally assessed by a general practitioner (GP), and if needed, referred to an orthopedic surgeon (OS) for a consultation, further management and possible surgical intervention [1]. Due to the increased number of patients with OA, orthopedic departments do not generally have the capacity to assess all patients referred from primary care in a timely manner. In addition, a large proportion of the patients assessed with OA are not considered eligible for surgical intervention, i.e. a total hip/knee replacement (THA/TKA) [2–4]. To increase access to secondary care, the clinical pathways for patients in need of a THA and TKA should be optimized [5]. Pathway optimization should also reduce direct and indirect costs, as well as increasing patient and societal satisfaction [2].

One way of optimizing these clinical pathways is to implement a physical therapy-led (PT-led) orthopedic triage care model. PT-led orthopedic triage means that patients with orthopedic disorders are assessed for the most suitable care by a physical therapist (PT) [6, 7]. A recent systematic review suggests, with low to moderate evidence, high agreement between PTs and OS treatment approach and diagnosis, as well as higher surgery conversion rates with PT-led orthopedic triage in patients with musculoskeletal disorders [8]. Furthermore, perceived quality of care with PT-led triage was higher or equal compared to standard care. The authors conclude that there is a low number of RCTs’ and that existing studies are of variable methodological quality. A previous Swedish study of PT-triage of patients with musculoskeletal disorders in primary care reported that this model of care can be effective for surgery conversion rate, shorter waiting times [9], with good patient perceived quality of care [10] and no difference regarding short or long term patient-reported outcomes [9]. Patients’ satisfaction with care after PT-led triage has been explored in previous studies among patients with musculoskeletal disorders and PT-led triage has been reported to provide patients with appropriate care [6, 11, 12]. It has also been reported that a PT-led orthopedic triage care model possibly could reduce the costs [13]. Appointments and assessments with a PT are significantly cheaper than appointments and assessments with an OS. According to reports from four different countries. The cost of visiting an OS is nearly twice as much as a consultation by an advanced practice PT [14–20].

When introducing a new care model, it is important to evaluate the patient’s perception of quality of care [21]. A good care model is characterized by being patient-centered, including respectfulness and individualized care [5, 22, 23]. Allowing the patients to participate actively in the decision-making process relating to their care is a key component in a patient-centered care model. Wilde and colleagues [21] created a model that represents the patients’ perceived quality of care based on their encounters with existing care structures, their norms, expectations and experience. Based on this model, the Quality from the Patient’s Perspective (QPP) questionnaire was developed [21]. The QPP is a validated and reliable questionnaire that measures patients’ perception of care and has been used in previous studies when evaluating patients’ experiences of and satisfaction with given care [21].

To our knowledge, no previous high-quality study has evaluated PT-led orthopedic triage in patients with hip or knee OA in a secondary care setting. Therefore, the aim of this prospective, randomized, controlled study was to compare perceived quality of care after PT-led triage with standard practice in a secondary care setting, for patients with primary hip or knee OA. The primary outcome was the statement “I *received the best possible examination and treatment”*. The hypothesis was that there would be no difference between groups regarding patients’ perceived quality of care.

## Methods

### Study setting and participants

Patients with primary hip or knee OA, referred from primary care for an orthopedic assessment at Sahlgrenska University Hospital between October 2020 and February 2022, were asked to participate in the study. The following inclusion criteria were used: primary hip or knee OA and ability to understand written and spoken Swedish. Patients were excluded if they (a) had previously been assessed by an orthopedic surgeon for their hip or knee OA, (b) had secondary OA, i.e. femoral head necrosis, or (c) were referred to a specific orthopedic surgeon. Ethical approval was obtained (registration number 2020 − 01144) from the Swedish Ethical Review Authority prior to the initiation of the study. The trial is registered at Clinical Trials NCT04665908, registered 14/12/2020.

### Procedure

Patients were randomized after providing verbal informed consent to participate over the phone. The randomization list was generated using an online randomization program (randomization.com). The informed written consent was obtained by post after randomization. The patients subsequently received appointments at the hospital by post. If the hospital was not able to provide the patient with an assessment within a reasonable time (in Sweden, this is considered to be 90 days), the patients were offered an assessment at another hospital. The patients were still able to continue their participation in the study and, depending on which hospital they were sent to, either the patient or the administrative staff at the hospital informed the first author (LG) about the date of their assessment. Due to the nature of the study, it was not possible to blind either the caregivers or the patients.

### Physical therapy-led orthopedic triage

Three physical therapists (PTs) with long experience (20–40 years) of working with patients with OA and about one year’s experience of the PT-led triage of patients with OA conducted the physical assessment in this trial. The duration of the appointment was set at a maximum of 60 min, with the aim of determining whether the patient was a candidate for a total hip/knee replacement (THA/TKA). The assessment was performed according to a checklist created by a team consisting of the three PTs, two OS and the research group. The checklist was used to ensure that all relevant information needed to decide on the patient’s suitability for surgical intervention were obtained. The checklist consisted of questions pertaining to the patient’s pain and disabilities, as well as details of a full clinical examination. During the assessment, the PTs also provided the patient with general information about OA and advice on how best to handle their OA with physical activity. If the patient was assessed as not being a candidate for surgery, they received the information about this and continued care during the assessment. If the assessment was unclear, or if the patient was considered suitable for surgery, the PT informed the patients about this and the fact that the assessment would be discussed with the OS before making a decision. The patients also received information that they would receive a letter at home with the final decision about continued care. All patients in the PT-led triage group were discussed during a weekly conference with the assessing PT and an assigned OS. Prior to the conference, the OS was provided with the checklist and the X-ray images. Based on this, blinded to the PT’s decision, the OS assessed whether the patient was suitable for surgery, non-surgical treatment, or a follow-up visit. During the conference, the PT informed the OS about the findings from the assessment according to the checklist. Both the PT and the OS then informed one another about their decision and together they made a final decision about the patient’s suitability for surgery. After the conference, a letter containing the final decision about continued care, i.e. whether the patient was suitable for surgery, non-surgical treatment or a follow-up visit with an orthopedic surgeon, was sent to the patient. The follow-up with an OS could be either a phone call or an appointment at the hospital and was scheduled within four weeks or up to six months after the initial assessment.

#### Standard care

The patients in the standard care group were assessed by one of the physicians, either an OS or a resident at the arthroplasty section of the orthopedic department. The duration of the appointment was set at a maximum of 30 min, with the aim of determining whether the patient was a candidate for THA/TKA. The patient received a decision on continued care from the physician during their visit, i.e. whether they were suitable for surgical treatment or non-surgical treatment. If needed, a follow-up, by either a phone call or an appointment at the hospital, was scheduled within four weeks or up to six months after the initial assessment.

### Outcome measurements

A short form of the Swedish version of the QPP questionnaire [21] was used to assess the patient’s perceived quality of care. The QPP has been psychometrically tested and validated in different settings [21]. The QPP is divided into four dimensions, however the present study only included three dimensions, *caregivers medical-technical competence*, *identity-oriented approach* and *outcome-related aspects of quality of care*. In total, 11 questions from the three dimensions were used in the study (one from *medical-technical competence*, seven from the *identity-oriented approach* and three from the *related aspects of quality of care*). The primary outcome was the statement “I *received the best possible examination and treatment”.* The fourth dimension, *physical-technical conditions*, were not used due to that the questions was not relevant for this study. The questions are answered in two ways using a 4-point Likert scale. The patient first rates how they perceive their quality of care (PR = perceived reality), *“This is what I experienced…”* and then how important that aspect of care is (SI = Subjective importance), *“This is how important this is to me…”*. The PR is rated from 1 (completely agree) to 4 (do not agree at all) and the SI from 1 (of very high importance) to 4 (little to no importance). Each item also has a *“Not applicable”* response option. Two questions in the QPP were included as secondary outcomes, *“Will you follow the advice of the physical therapist/orthopedic surgeon?”* (response options 1 [No] to 3 [Yes, completely] and not applicable [*“Don’t know”* or *“I have not received advice/information”*]) and *“To what extent were your expectations of the treatment met?”* (rated on a 5-point Likert scale ranging from 1 [Not at all] to 5 [To very large extent]).

The questionnaire was sent to each patient (either by post or as an online survey as decided by the patient) within a week of their visit to the orthopedic clinic. Up to four reminders (within three weeks) were sent by email/post and finally one reminder by phone. Improve IT (Halmstad, Sweden) distributed and administered the online survey questionnaire. The first author of the study (LG) sent the questionnaire by post.

Demographic data including age, gender, civil status, country of birth and education level were collected at baseline to describe the study population.

### Sample size

A power analysis was calculated for the clinical trial. A relevant mean difference between groups for the statement *“I received the best possible examination and treatment”* on the QPP was suggested to be 0.35 [10, 24]. Sample size calculation was performed with G*power software (G*Power-Free download and software reviews – NET Download), which gave a total group size of 378 patients to detect a mean difference of 0.35 in the QPP with 95% power, a significance level of 0.05 and a standard deviation of 0.94. Initially, the project estimated a dropout of 20%. Due to the Covid-19 pandemic and the inability of the hospital to provide all randomized patients with appointments, it was later decided to increase the estimated dropout to 50% and 764 patients were therefore randomized.

### Data analysis

Descriptive statistics for demographics were analyzed using the Mann-Whitney U test to determine any baseline differences. Between-group comparisons from the QPP were performed with the Mann-Whitney U test; median values were also reported. The significance level was set at p < 0.05. All the collected data were transferred and analyzed by the first author (LG) using IBM SPSS version 28 (IBM, Corp, Armonk, NY, USA).

## Results

### Participants

**Fig. 1:**
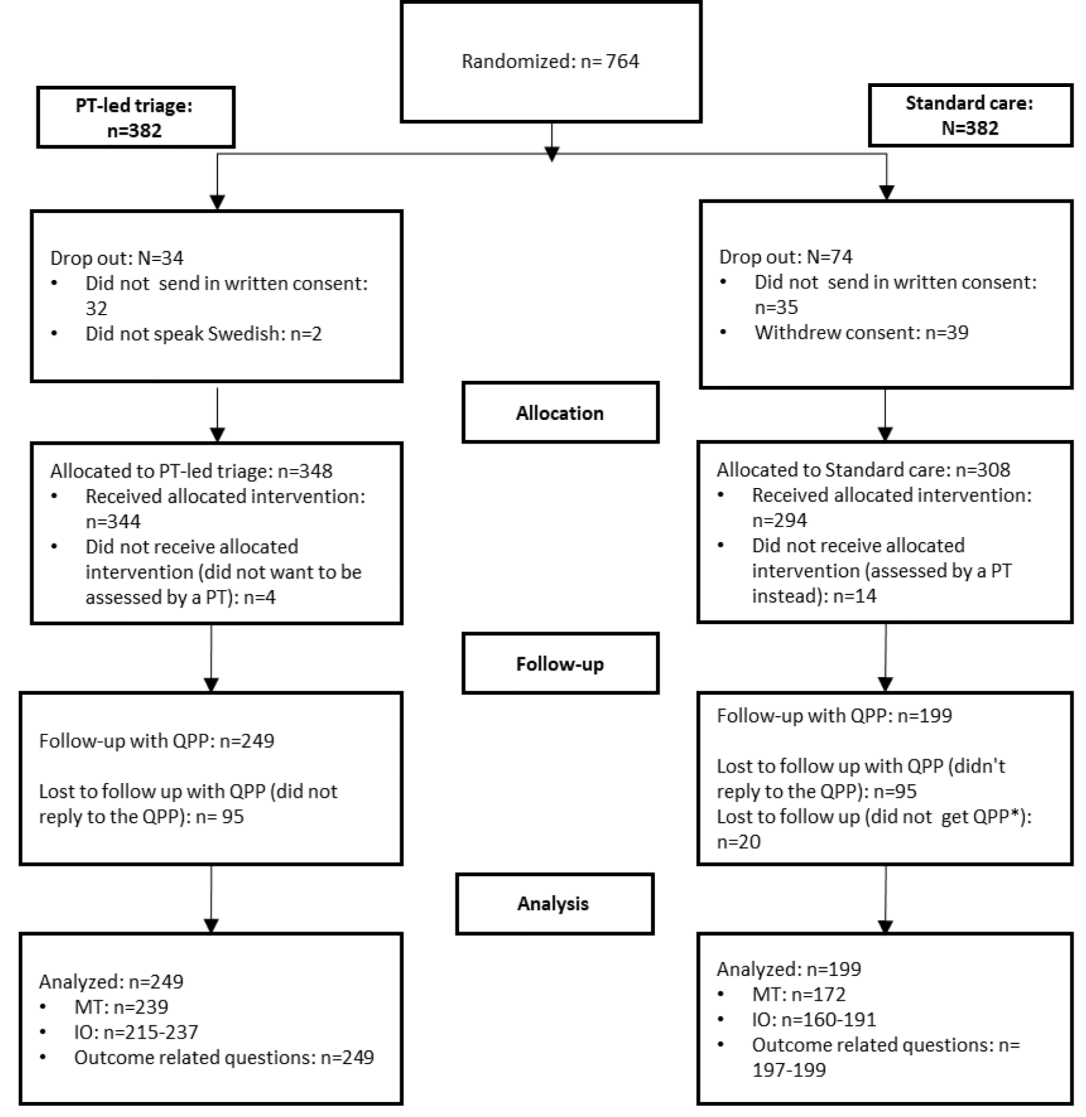
Flowchart of the progress of participation in the study. PT = physiotherapist, QPP = Quality from the Patients’ Perspective, MT = medical-technical competence, IO = Identity-Oriented approach

A total of 764 patients with primary hip or knee OA agreed to participate (Fig. 1). However, only 656 patients gave their written consent to participate in the study and, as a result, 108 patients were excluded before the start of the intervention. Fourteen patients in the standard care group were mistakenly assigned to an assessment by PT triage and were therefore excluded from the study. Some patients in the standard care group (n = 20) that were assessed at another hospital did not provide the first author (LG) with the date of their assessment and, for this reason, they did not receive the QPP and were excluded. A total of 121 patients (33 in the PT group, 88 in the standard care group) were excluded before the intervention. The QPP was sent to 638 patients and the overall response rate for the QPP was 70% (72% in the PT-led triage group and 67% in the standard practice group). The reason for non-responders is unknown. Patient demographics can be found in Table 1.

**Table 1:** Demographic characteristics of the respondents of the QPP questionnaire at baseline

	Physiotherapy-led triage (n = 249)	Standard care (n = 199)
*Age (years)*		
Mean (SD)	69.2 (9.7)	68.9 (9.1)
Min-max	42–90	49–91
*Sex*		
Female	156 (62)	120 (61)
*Civil status*		
Married/living together	156 (63)	114 (57)
Single/living alone	84 (34)	71 (36)
Missing	9 (3)	14 (7)
*Country of birth*		
Sweden	218 (87)	165 (83)
Other Nordic countries	9 (4)	9 (4)
Other country in Europe	7 (3)	8 (4)
Country outside Europe	7 (3)	3 (2)
Missing	9 (4)	14 (7)
*Education*		
Elementary school	36 (15)	26 (13)
Upper secondary school	91 (36)	74 (37)
University	114 (46)	85 (44)
Missing	8 (3)	12 (6)
*Occupation*		
Working	66 (27)	56 (28)
Retired	176 (70)	132 (66)
Other	7(3)	9 (4)
Missing	1 (0)	2 (1)

QPP = Quality from Patients’ Perspective; SD = Standard deviation. All

variables are presented as counts (n) and frequencies (%) or mean and SD

Statistically significant differences between groups were analyzed with the Mann-Whitney U test. The statistically significant level was set to < 0.05. No difference in demographic characteristics were found between groups

### Perceived reality

No significant difference was found between the groups (*p* = 0.6) regarding the primary outcome statement *“I received the best possible examination and treatment”*. Furthermore, the PT-led triage group reported significantly better results in terms of receiving information on how they should take care of themselves (*p* = 0.0.17). Meanwhile, patients in the standard care group reported being more involved in the decision-making relating to their continued care (*p* = 0.005). The standard care group also perceived to a greater extent that their care was determined by their need for care, rather than staff routines, compared with the PT-led triage group (p = 0.007). Detailed data on the outcomes of perceived reality are presented in Table 2.

**Table 2:** Results from the questionnaire Quality from the Patients ‘Perspective (QPP).

Dimension/factor	Physiotherapy-led triage (n = 249)			Standard care (n = 199)				
	n	NA	Median /Q1;Q3	n	NA	Median /Q1;Q3	*p*-value	
**Medical-technical competence**								
*Care I received*								
I received the best possible examination and treatment								
PR	239	10	1/1;2	172	17	1/1;2		0.6
SI	239	10	2/1;2	172	17	2/1;2		0.42
**Identity-oriented approach**								
*Receiving information about*								
How examination and treatments would take place								
PR	237	12	1/1;2	189	10	1/1;2		0.57
SI	237	12	1/1;2	189	10	2/1;2		0.36
Self-care: “How I should take care of myself”								
PR	237	12	2/1;2	166	33	2/1;3		**0.017**
SI	237	12	2/1;2	166	33	2/1;3		0.43
*Participation I decision making*								
I had the opportunity to participate in decision								
PR	215	34	2/1;3	183	16	1/1;2		**0.005**
SI	215	34	1/1;2	183	16	1/1;2		**0.0016**
*Caregivers’ understanding, commitment and respect*								
Seemed to understand how I experienced my situation								
PR	246	3	1/1;2	191	8	1/1;2		0.83
SI	246	3	1/1;2	191	8	1/1;2		0.67
Was respectful towards me								
PR	247	2	1/1;1	192	7	1/1;1		0.56
SI	247	2	1/1;1	192	7	1/1;1		0.88
Showed commitment: cared about me								
PR	247	2	1/1;1	191	8	1/1;1		0.78
SI	247	2	1/1;2	191	8	1/1;2		0.97
Care according to staff routines								
PR	215	34	2/1;3	160	39	1/1;2		**0.007**
SI	215	34	1/1;2	160	39	1/1;2		0.1

Item scores for medical-technical competence and identity-oriented approach on the questionnaire Quality from the Patients ‘Perspective (QPP). Response option for perceived reality (PR ranged from 1 (“Completely agree”) to 4 (“Do not agree at all”) and subjective importance (SI) from 1 (“Of the very highest importance”) to 4 (“Little or no importance”). NA: Not applicable. Q1;Q3: First quartile; third quartile. Statistically significant differences between groups (analyzed with the Mann-Whitney U test) are presented in bold. The statistical significant level was set to < 0.05

### Subjective importance

Significant difference were found in one of the questions regarding subjective importance (SI) (Table 2). Patients in the standard care group considered it was more important to be involved in the decision-making process relating to their continued care than those in the PT-led triage group (*p* = 0.016). The two groups reported equal subjective importance in the other eight questions (Table 2).

### Outcome-related aspects

The standard care group reported that their expectations of the assessment were met to a significantly greater extent (*p* = 0.013; Table 3). Meanwhile, the PT-led triage group reported that they would follow the advice and instructions they received to a greater extent than the standard care group (*p* = 0.05). In addition, both groups were positive about visiting the clinic again in the future, if needed.

**Table 3:** Outcome-related aspects of quality of care

	Physiotherapy-led triage (n = 249)			Standard care (n = 199)			
Question	n	NA	Median/Q1;Q3	n	NA	Median/Q1;Q3	*p*-value
Meeting of expectations	249	0	2/1;2	197	2	1/1;1	**0.013**
Intention to follow advice and instructions	249	0	1/1;2	198	1	1/1;2	0.05
Wanting to visit this clinic in the future	249	0	1/1;1	199	0	1/1;1	0.6

Item scores for outcome-related aspects on the questionnaire Quality from the Patients ‘Perspective (QPP). Response options ranged from 1 (“To very large extent”) to 5 (“Not at all”) for the item regarding expectations, from 1 (“Yes, completely”) to 3 (“No”) for the item regarding intentions, and from 1 (“Yes, absolutely”) to 3 (“No”). NA: Not applicable. Q1;Q3: First quartile; third quartile. Statistically significant differences, p < 0.05, between groups (analyzed with the Mann-Whitney U test) are presented in bold. The statistical significant level was set to < 0.05

## Discussion

To our knowledge, this is the first study comparing patients’ perceived quality of care after PT-led triage with standard care in a secondary care setting for patients with hip or knee OA. The perceived quality of care was assessed with a short form of the QPP within a week after the examination at the orthopedic clinic. Overall, patients in both groups reported good quality of care. However, significant differences between the groups were found on some questions on the QPP, one in favor for the PT-led triage group and three in favor for the standard care group.

Before implementing a new care model at the clinic, it is important to evaluate the model’s suitability. A good care model is characterized by being patient-centered [21] and it is therefore important to evaluate patients’ perception of quality of care. In the present study, both groups reported similar perceived quality of care regarding the primary outcome *“I received the best possible examination and care”*, which is in line with other studies reporting a high level of patient satisfaction after PT-led triage [10, 11, 25, 26]. The hypothesis was that there would be no difference between the groups regarding patients’ perceived quality of care. The result for this question strengthened the hypothesis and indicates that patients feel that PT-led orthopedic triage is as good as standard care regarding examination and care. This is in line with a qualitative study by Reeves et al. [11] of patients’ perspectives of the quality of a PT spinal screening and service, reporting that patients expect both PTs and OS to be appropriately qualified in terms of examination and treatment. It is important that patients feel satisfied and have confidence in their caregivers’ knowledge. If not, the patients will again seek care with other caregivers, which will increase the cost to both the healthcare system and society. The result of the primary outcome suggests that a PT-led triage model according to patients’ perception can be used while maintaining an appropriate level of care.

One of the PT’s main tasks, in both primary and secondary care, is to give patients advice on how to best handle their daily activities and to recommend appropriate physical activities. Information, exercise and pain relief are given to a greater extent during an assessment by a PT compared with other medical staff [4, 6, 10, 27]. As a result, it is not surprising that the PT-led triage group reported that they received better advice on how to take care of their OA. Since the majority of the patients referred for an orthopedic assessment are not suitable for surgical intervention [2], it may be more important for the patient’s well-being to receive information on how to take care of their OA. However, to minimize the waiting time for an assessment at the orthopedic clinic in the future, some of these patients need to be identified at an earlier stage and to be re-sent to primary care for continued care before an assessment at the orthopedic clinic.

The standard care group reported having more opportunity to participate in the clinical decision-making progress relating to their continued care. One of the OS’s main tasks is to determine whether a patient is suitable for surgical intervention and subsequently perform the surgery if needed. This makes it possible for the OS to be more flexible during their assessment, to discuss the different treatment options with the patient to a greater extent and, together with the patient, decide on the best option. In the present study, in the PT-led triage group, only patients who were evidently not suitable for THA/TKA received the decision during the assessment meeting. The remaining patients did not receive the decision during the assessment meeting but instead had to wait for a letter with the final decision about the most suitable care and they were therefore not able to participate in the final decision-making process. In most cases, the patients received the decision after they had already submitted the QPP. This may explain the difference in the question regarding the opportunity to participate in the clinical decision-making process. As mentioned, a key component of a good care model is involving the patient in the decision-making process relating to their care [21–23]. In the present study, the PTs who performed the assessments always asked the patient if he/she was interested in surgical intervention. However, PTs were not able to make final decision about surgical interventions and including the patient in the final decision-making might therefore have been challenging. One way of including the patient in the decision-making process to a greater extent could be to inform the patient about the final decision over the phone, which would then make it possible for them to ask questions about the decision and feel more included in the process. It could also be of value if the final decision could be delivered during the consultation with the PT.

Expectations of the visit were met to a greater extent in the standard care group. When consulting a caregiver (PT or physician), it has previously been reported that the most important expectation is not to recover but to have their disorder confirmed [28–30]. In the present study, all the patients had knowledge of their diagnosis prior to being referred for an orthopedic assessment. In a previous study by Samsson et al. [10], it was reported that PT-led triage met patients’ expectations to a greater extent. The difference in outcomes between the present study and the one by Samsson et al. [10] might be explained by the difference in the care models/process, the different study populations and the number of patients included (348 patients versus 203 patients). As has previously been addressed, the perceived lack of influence on the decision-making might also have affected the expectation outcome. During the PT triage, the patients also received information on self-care and physical activity. As it is usual for patients in primary care to receive this prior to a referral for an orthopedic assessment, the patients might feel that they did not receive any new information, which may have influenced the outcome. Considering, however, that the majority of the patients referred for an orthopedic assessment have not received the appropriate treatment for OA and are therefore not eligible for surgical intervention [2], it can still be argued that most of the patients could benefit more from an assessment by an PT.

Overall, patients in both groups perceived good quality of care. For example, patients in both groups were positive about visiting the clinic again in the future, if needed. Furthermore, both groups said that they received information to a greater extent about how examinations and treatments would take place, together with the results of the examinations and treatments. Previous studies of a PT-led triage care model report a high level of agreement on diagnosis and treatment approach [4, 12, 31] and positive results regarding patients’ perceived quality of care. The result of the present study indicates that a PT-led orthopedic triage care model can be used in a secondary care setting for patients with primary hip or knee OA, while maintaining good quality of care. However, the care process could be optimized to include the patients to a greater degree in the decision-making process and to fulfill the patient’s expectations to a greater degree. Furthermore, it is important to educate the patients regarding the various levels of care process of OA and when and what type of care is appropriate.

### Methodological considerations

Only a few studies of the quality of care have used a randomized controlled design. This study also had a large sample of participants, sufficient power and is original, which, taken together, is a major strength. The fact that the assessment was made by different physical therapists and orthopedic surgeons can also be regarded as a strength, as it reflects the normal healthcare system and increases the generalizability of the findings.

The questionnaire used in this study has good validity. As a review of patient satisfaction with healthcare reported a mean response rate of 67% for questionnaires [32], the total response rate for the QPP in the present study of 70% should be regarded as good. However, the number of respondents differed between the PT-led triage (n = 249, 72%) and standard care groups (n = 199, 67%). It is possible that this could have influenced the results to produce either smaller or larger differences between the groups. In addition, when performing multiple testing there is always a risk of type I error.

The duration of the assessment was set according to the hospital’s standard practice and it therefore differed between the groups (PT triage 60 min, standard care 30 min). However, the longer duration of the PT-led triage assessment may have affected the outcome, as previous studies have reported that an adequate time may be a determinant of satisfaction [33].

The PTs was judged to be consistent regarding the decision about continued care for the patients due to the checklist. However, the physicians making the assessment in the standard care group, did not have a standardized protocol to follow during the assessment. Therefore, there might be differences regarding the physicians’ decisions on patients’ suitability for surgical intervention.

There is no data available on how many patients in the standard care group that were deemed to be eligible for surgical intervention during the initial assessment. If there were a difference between the groups regarding number of patients assessed to be a surgical candidate between the groups, it might have influenced the result.

Due to the covid-19 pandemic, the expected dropout was changed from 20 to 50%. Some patients did not wish to visit the orthopedic clinic due to the covid-19 pandemic and they therefore withdrew their verbal consent to participate in the study before the start of the intervention. The covid-19 pandemic also increased the waiting time for an orthopedic assessment by an OS due to fewer OS working at the clinic. As a result, some patients withdraw their consent due to the long waiting time (n = 39). In addition, more patients than usual were sent to other hospitals for an assessment and some patients (n = 20) were lost because the first author (LG) was not informed about the date of the assessment. Since the dropouts/exclusions were larger in the OS group than the PT-led triage group (n = 88 versus n = 38), this might have influenced the results.

### Future research

Patients’ participation in their care is a key component for a good patient-centered care model. Future research should therefore focus on ways of including PT-led triage patients to a greater degree in the decision-making progress relating to their care. This can preferably be done through qualitative research involving both patients and clinicians (PTs and OS). Qualitative studies could also assess ways of increasing the meeting of expectations in the PT-led triage group. It could also be relevant in future research to investigate if and how the number of hip and knee replacement surgeries change with the introduction of a PT-led triage care model as well as the patient’s pain and disabilities and health related quality of life. Finally, future research should also aim to investigate the cost-effectiveness and long-term follow-up after PT-led triage compared with standard care.

## Conclusion

The present study reports that patients in both the PT-led triage group and the standard care group perceived good quality of care. No difference was found between the groups for the primary outcome *“I received the best possible examination and treatment”*. Even though patients in the standard care group perceived that they participated to a greater extent in the decision-making process, as well as meeting expectations of their care, the PT triage model of care appears to provide an opportunity to give an appropriate level of care while maintaining good quality of care. However, due to the large number of dropouts, the result should be interpreted with caution.

## Electronic supplementary material

Below is the link to the electronic supplementary material.

Supplementary Material 1

## Data Availability

Due to General Data Protection, the data sets generated and analyzed during this study are not publicly available. However, they can be made available by the corresponding author in response to a reasonable request.
