# Supplementary material for: No major difference in perceived quality of care in patients with hip or knee osteoarthritis assessed in a physical therapy-led triage compared with standard care: a randomized controlled trial
Source: BMC Musculoskelet Disord. 2023 Jun 29;24:530. doi: 10.1186/s12891-023-06659-5 (PMC10308718; doi:10.1186/s12891-023-06659-5)
Supplement: Supplementary file 1 — Supplementary Material 1 [file 12891_2023_6659_MOESM1_ESM.pdf]

2020-06-03

Assessment checklist: PT-led orthopedic triage

Name: ____

Personal number: _________________________________

Pain medication	Yes	No	

If yes, which medication/how often? ________________________

Most painful joint:	R Knee		L Knee
	R Hip		L Hip
X-ray verified OA in most painful joint	Yes	No	
Joint stiffness	Yes	No	Sometimes
Night pain	Yes	No	Sometimes
Trusts knee/hip	Yes	No	Sometimes

Maximal walking distance: ______________with/without walking aid

Able to walk in stairs	Yes	No	
If yes, is it painful?	Yes	No	Sometimes

Exercise/physical activity	Yes	No	

What kind of activity? _________________________________

How often/duration? _________________________________

Tried rehabilitation:	Yes	No	

If yes, when? ______________________________________

For how long period of time? __________________________

Participated in BOA:	Yes	No	

Physical examination

K- Swelling in most painful joint? Yes No NA

K/H- Can stand on one leg: Yes Yes, but barely No

K- Joint stability Good stability Instable

ROM Hip/Knee:

K/H - Flexion_______________degrees

K/H - Extension_____________degrees

H – Hip abduction ___________degrees

H - Internal rotation Normal Limited Much limited

H – External rotation Normal Limited Much limited

Muscle function:

K/H – Can do a straight leg raise Yes No

K/H – Sit to stand Yes Yes, with help from the arms No

Pain from other joints? /Which joints? ____

Want surgical intervention? Yes No

Patients goal with surgical intervention ____

Decision

Is the patient suitable for THA/TKA?

Yes **No**
